# Pregnancy outcomes following oral brivudine exposure: two case studies with long-term follow-up

**DOI:** 10.1007/s00210-026-05154-7

**Published:** 2026-03-04

**Authors:** Nusret Uysal, Ismail Yilmaz, Baris Karadas, Nihal Olgac Dundar, Yusuf Cem Kaplan

**Affiliations:** 1 Department of Medical Pharmacology, Marmara University School of Medicine, Istanbul, Turkey; 2 TERAFAR (Izmir Kâtip Celebi University Teratology Information, Training and Research Center), Izmir, Turkey; 3 Department of Pediatrics, Division of Pediatric Neurology, Izmir Kâtip Celebi University, Izmir, Turkey

**Keywords:** Brivudine, Pregnancy, Congenital abnormalities, Teratology

## Abstract

Brivudine is a thymidine analogue antiviral agent used in the treatment of herpes zoster; however, human data regarding its use during pregnancy are lacking. This report aims to describe pregnancy outcomes following oral brivudine exposure and to provide the first human clinical data on brivudine use during pregnancy. Two pregnant women exposed to oral brivudine during the first and second trimesters were referred for teratological risk assessment and followed with perinatal monitoring and postnatal clinical follow-up. Both pregnancies were continued with perinatological follow-up and resulted in the delivery of healthy infants without major congenital malformations. Postnatal follow-up identified mild allergic manifestations in one child and an isolated mild delay in the personal–social developmental domain in the other. These cases provide preliminary clinical data on brivudine exposure during pregnancy and contribute to the limited human data available.

## Introduction

Brivudine is a thymidine analogue antiviral agent with potent activity against varicella-zoster virus (VZV) and herpes simplex virus type 1 (HSV-1) (Prusoff 1959). It is selectively phosphorylated by the viral thymidine kinases of HSV-1 and VZV, leading to the formation of its active triphosphate metabolite, which inhibits viral DNA polymerase and suppresses viral DNA synthesis (De Clercq and Li 2016). Brivudine is approved in many countries worldwide, including Turkey, for the oral treatment of herpes zoster at a dosage of 125 mg once daily for 7 days (De Clercq and Li 2016).

A systematic review of comparative treatment trials involving 5872 patients with HSV infections reported that a 14-day course of brivudine was at least as effective as acyclovir and ganciclovir (Wilhelmus 2010). Brivudine is also available in ophthalmic formulations for HSV-1 epithelial keratitis, and it has been reported as a potential therapeutic option in Epstein–Barr virus encephalitis (Lahmer et al. 2010). Current clinical guidelines do not specifically address brivudine use during pregnancy, and in the absence of human safety data, nucleoside analogues with established pregnancy safety profiles, such as acyclovir, are generally preferred (Briggs et al. 2021). This lack of evidence creates a clinical dilemma when inadvertent brivudine exposure occurs during pregnancy.

To our knowledge, there are currently no published human data regarding brivudine exposure during pregnancy. In this context, the present case report provides novel clinical data by describing two pregnant women exposed to oral brivudine during the first and second trimesters and their pregnancy outcomes. These cases were previously presented in preliminary form, and the present study provides expanded clinical information, detailed evaluation, and extended follow-up that were not included in the initial report.

## Methods

Two pregnant women exposed to drugs during pregnancy, with no significant medical history and taking folic acid (400 mcg/day), were referred to TERAFAR for risk assessment. Informed consent for the use of clinical information for publication was obtained from both patients. Clinical data regarding drug exposure, concomitant medications, pregnancy course, delivery outcomes, and postnatal follow-up were evaluated. Neurodevelopmental assessment was performed using the Denver Developmental Screening Test, and subsequent clinical follow-up information was obtained. In both cases, drug exposure occurred prior to pregnancy recognition, and treatment decisions were made by the primary physicians before referral. The patients were subsequently referred to TERAFAR for post-exposure teratological risk assessment and counseling.

## Results

### Case 1

At gestational week 4, the first case (a 27-year-old, 6-week pregnant woman) received brivudine tablets (125 mg/day) for 7 days for herpes zoster, as well as fusidic acid topically for 4 days and a single dose of oral vitamin D3 solution (300,000 I.U./day). After receiving teratological counseling at TERAFAR, the mother decided to continue the pregnancy with perinatology follow-up and gave birth to a healthy baby via cesarean section.

No birth defects were diagnosed in the first case, and the baby’s physical and neurological development was normal at the age of one. Neurodevelopmental evaluation at 1 year of age using the Denver Developmental Screening Test showed age-appropriate development across all domains. The infant’s medical history was unremarkable except for a mild allergy. After immunologic evaluation, general recommendations were given, and no treatment was recommended.

### Case 2

The second case (a 19-year-old, 16-week pregnant woman) declared that she had used brivudine tablets (125 mg/day) for 7 days for herpes zoster at the 15th week of her pregnancy. The patient also used topical 1% diphenhydramine HCl lotion, paracetamol, and oral ferrous fumarate. After receiving teratologic counseling, the mother continued the pregnancy with perinatology follow-up and gave birth to a healthy baby.

Similarly, no birth defect was diagnosed in the second case. During the neurodevelopmental evaluation at age 1, a delay was detected in the personal–social domain using the Denver Developmental Screening Test. Subsequent follow-up showed that the child demonstrated age-appropriate development at approximately 3.5 years.

## Discussion

Brivudine is a thymidine analogue antiviral agent that requires phosphorylation by viral thymidine kinases to exert its antiviral activity, resulting in selective inhibition of viral DNA polymerase (De Clercq and Li 2016). This general mechanism of selective viral activation and inhibition of viral DNA synthesis is shared by other nucleoside analogues, such as acyclovir, valacyclovir, and famciclovir (Pasternak and Hviid 2010). Data from the Acyclovir/Valacyclovir Pregnancy Registry, established by the manufacturer, reported no increase in congenital defects among 1246 acyclovir-exposed pregnancies, including 756 first-trimester exposures, and among 56 valacyclovir-exposed pregnancies, 14 of which involved first-trimester exposure (Reproductive Toxicology Center 2026). The absence of any birth defects in both infants described in this report is therefore consistent with the pharmacological profile of nucleoside analogues and the available safety data from related antiviral agents.

In the first case, mild allergic manifestations were observed during infancy. Atopic conditions are among the most common chronic disorders in childhood, with prevalence estimates of up to approximately 40% in pediatric populations (Pierau et al. 2021). Based on a single observation and in the absence of a plausible biological mechanism linking brivudine exposure to allergic disease, no causal association can be established. This finding should therefore be interpreted with caution.

In the second case, a neurodevelopmental assessment at age 1 using the Denver Developmental Screening Test revealed a mild delay confined to the personal–social domain. Developmental screening tools such as the Denver test are intended for early identification of potential concerns rather than for definitive diagnosis. Isolated findings limited to a single developmental domain, particularly in the absence of abnormalities in other domains, should be interpreted with caution.

Both pregnancies were managed with teratological counselling and close perinatological follow-up, highlighting the importance of individualized risk assessment and shared decision-making when drug exposure occurs during pregnancy. These cases illustrate how structured counseling and monitoring may support informed continuation of pregnancy in situations where human safety data are scarce.

Deriving causality and definitive conclusions from case reports is inherently limited, especially in the context of drug exposure during pregnancy; nonetheless, these cases contribute incremental information to the scarce safety data available prior to epidemiological evaluation.

## Conclusion

These cases provide the first human clinical data on pregnancy exposure to brivudine. In both pregnancies, no major congenital malformations were observed, and follow-up findings were generally unremarkable. Further data are needed to better characterize the safety profile of brivudine during pregnancy.

## Data Availability

All source data for this work (or generated in this study) are available upon reasonable request.
